# Invest in the future: “Hands-on Radiology” summer school

**DOI:** 10.1186/s13244-023-01382-0

**Published:** 2023-03-29

**Authors:** Laura Segger, Adrian A. Marth, Vitus Gosch, Jonas Oppenheimer, Sophia Lüken, Annika Bierbrauer, Martine S. Nilssen, Mona Jahn, Bernd Hamm, Markus Lerchbaumer, Timo A. Auer

**Affiliations:** 1 Department of Diagnostic and Interventional Radiology, Campus Virchow-Klinikum, Charité – Universitätsmedizin Berlin, 13353 Berlin, Germany; 2 Department of Radiology, Campus Benjamin-Franklin, Charité – Universitätsmedizin Berlin, 13353 Berlin, Germany; 3 Innlandet Hospital, Division Tynset, Sjukehusveien 9, 2500 Tynset, Norway; 4 Central Institute of Radiology (ZIR), Klinikum der Stadt Ludwigshafen am Rhein gGmbH, Bremserstr. 79, 67063 Ludwigshafen, Germany; 5 Department of Radiology, Campus Mitte, Charité – Universitätsmedizin Berlin, 13353 Berlin, Germany; 6 Berlin Institute of Health (BIH), Anna-Louisa-Karsch-Straße 2, 10178 Berlin, Germany

**Keywords:** Summer school, Medical students, Undergraduate radiology education, Radiology education, Radiology teaching

## Abstract

**Purpose:**

The field of radiology is currently underestimated by undergraduate medical students. The “Hands-on Radiology” summer school was established to improve radiology knowledge and interest among undergraduates. The purpose of this questionnaire survey was to analyze whether a radiological hands-on course is an effective tool to reach and motivate undergraduate students.

**Materials and methods:**

The three-day course held in August 2022 included lectures, quizzes, and small group hands-on workshops focusing on practical work with simulators. All participants (*n* = 30) were asked to rate their knowledge and motivation to specialize in radiology at the beginning of the summer school (day 1) and the end (day 3). The questionnaires included multiple choice questions, 10-point scale questions and open comment questions. The second questionnaire (day 3) included additional questions regarding the program (topic choice, length, etc.).

**Results:**

Out of 178 applicants, 30 students (16.8%) from 21 universities were selected to participate (50% female and 50% male students). All students completed both questionnaires. The overall rating was 9.47 on a 10-point scale. While the self-reported knowledge level increased from 6.47 (day 1) to 7.50 (day 3), almost all participants (96.7%, *n* = 29/30) mentioned an increased interest in the specialization of radiology after the event. Interestingly, most students (96.7%) preferred onsite teaching instead of online teaching and chose residents over board-certified radiologists as teachers.

**Conclusion:**

Intensive three-day courses are valuable tools to strengthen interest in radiology and increase knowledge among medical students. Particularly, students who already have a tendency to specialize in radiology are further motivated.

## Key points

1. The three-day student program enhanced the visibility of radiology as a specialization.
2. Interest in and knowledge of radiology was increased among participants.
3. Participants’ motivation to consider specialization in radiology increased.
4. Participants favored onsite teaching and teaching by residents instead of consultants.
5. Networking is a major factor during such courses among participants.

## Introduction

The German Association of Young Radiologists e.V. was founded in 2018 by radiology residents, mostly in their second or third year of residency, with the aim to gain more visibility, increase knowledge and interest in radiology, and potentially inspire students to specialize in radiology. Since radiology receives little attention in the national curriculum, there is a gap of knowledge about the wide field of radiology and its interdisciplinary relevance in patient care among undergraduate medical students [1]. A three-day “Hands-on Radiology” summer school was implemented to close this gap and demonstrate the practical role of radiology in terms of both diagnosis and interventional treatment. While quantitative data on applications for residency in radiology in Europe are sparse, Hoffmann et al. noted a decreasing interest over the last years in the USA, concluding that it is important to engage medical students in radiology to once again attract more candidates to this specialty [2]. Additionally, advances in artificial intelligence are lowering medical students’ interest to specialize in radiology since they fear that radiologists will be replaced by AI [3, 4]. Reports on a few summer schools in different fields for medical students suggest that such courses can strengthen students’ interest in the field [5–9]. Supported by the European Society of Radiology, the “Hands-on Radiology” summer school first took place in 2018 and again in 2019 and then paused due to the COVID-19 pandemic. The third summer school took place from August 25 to 27, 2022, and was surveyed in our study. The COVID-19 pandemic increased the tendency to switch to online education formats [10, 11]. The question arises if this is a trend that is recommendable for radiology education.

The aim of the survey presented here was to investigate whether a summer school in radiology is a suitable tool to improve radiology knowledge and strengthen interest in radiology in undergraduate medical students.

## Materials and methods

A total of 178 applications for 30 spots were received during a 3-month application phase (March–June 2022). The event was announced via the homepage of the German Association of Young Radiologists e.V. (https://www.young-radiologists.com), social media (Facebook, Instagram), and through sending a circular email to all German medical universities. Candidates had to complete an online form requesting some general information (name, semester, university) and a motivational letter. Exclusion criteria were first- and second-year medical students, due to their lack of clinical knowledge, as German medical students do not begin with clinical training until the third year, and licensed physicians. Participants were selected by a consensus vote of four members of the Association of Young Radiologists e.V. mainly taking the motivational letters into account. In case of a tie, remaining spots were filled paying special attention to gender equity and incorporation of students from different medical schools in Germany and neighboring countries. The “Hands-on Radiology” summer school took place at a German university clinic from August 26 to 28, 2022. The event was free of charge, and onsite catering and a social dinner for the first evening were organized. Travel and accommodation had to be organized by participants, these expenses were not reimbursed. Thirty participants from 21 universities in Germany and neighboring countries participated in the summer school.

**Fig. 1:**
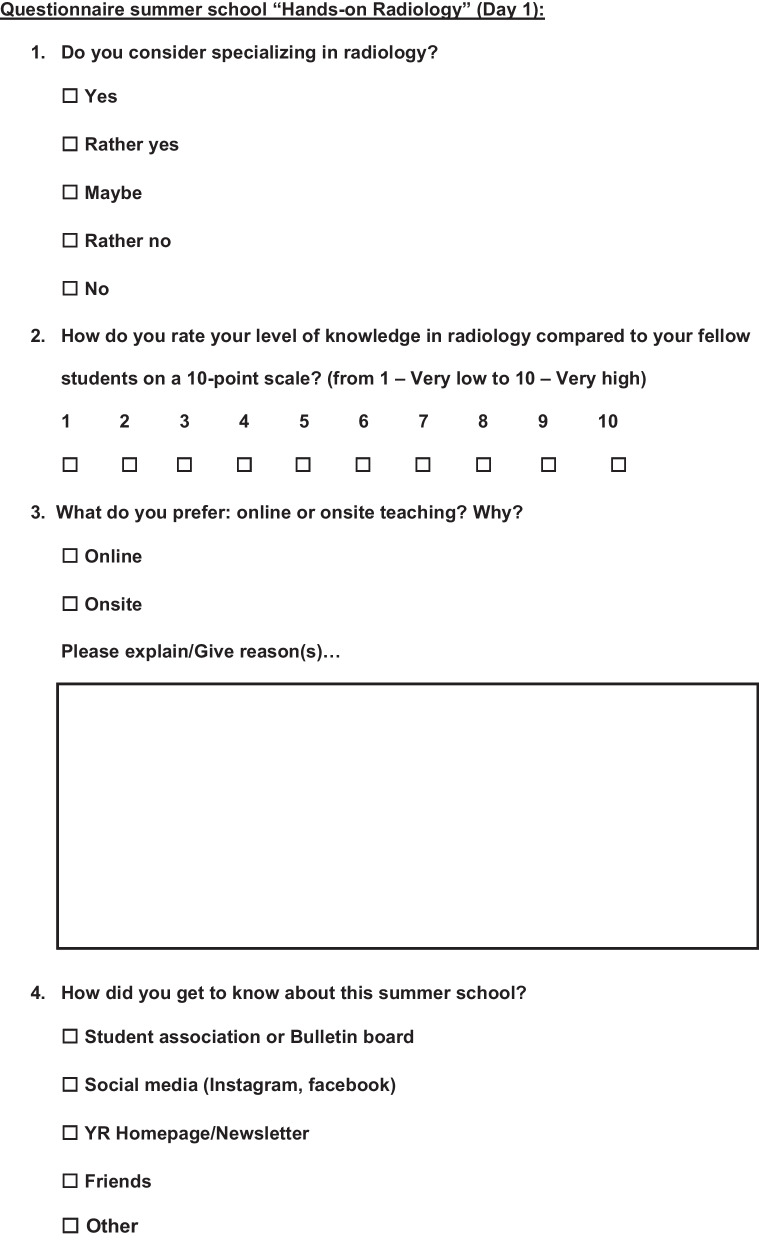

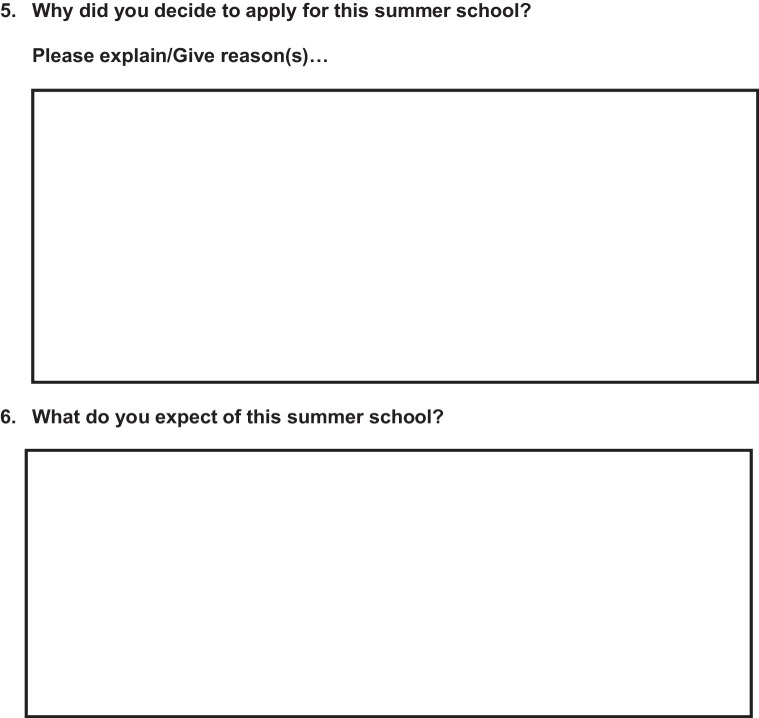
Questionnaire at the beginning of the Summer School

**Fig. 2:**
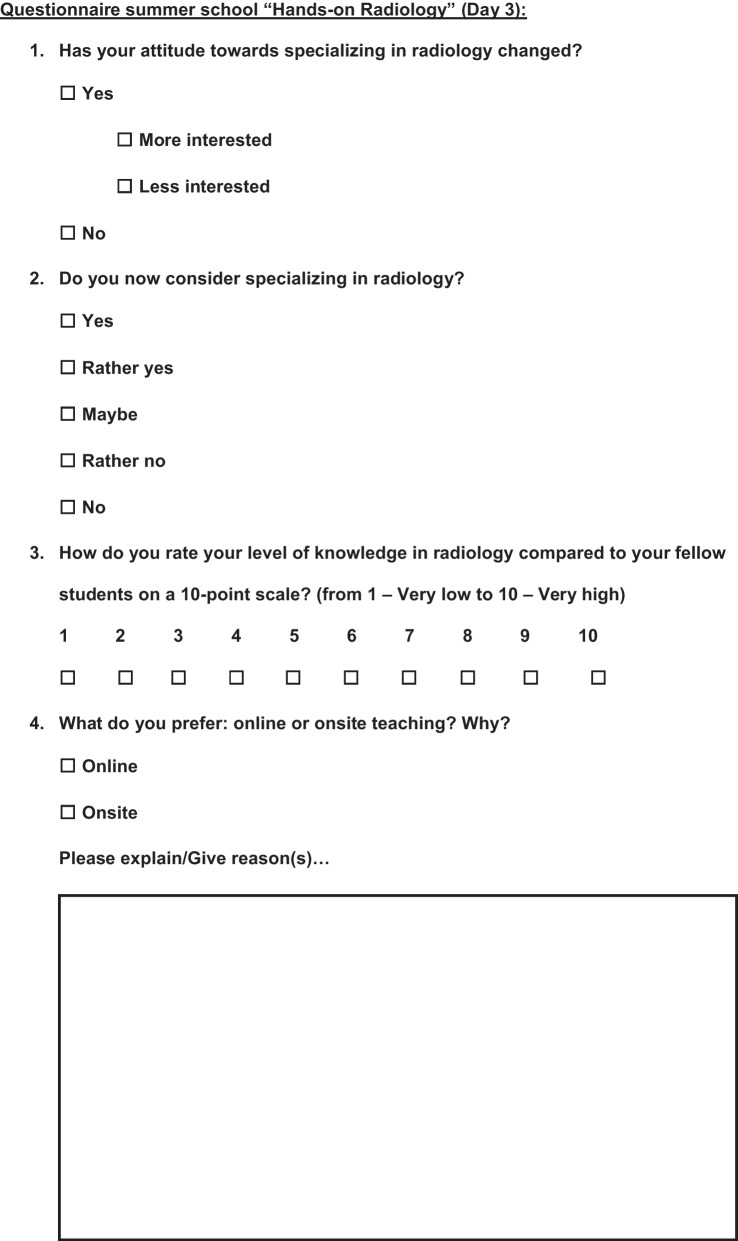

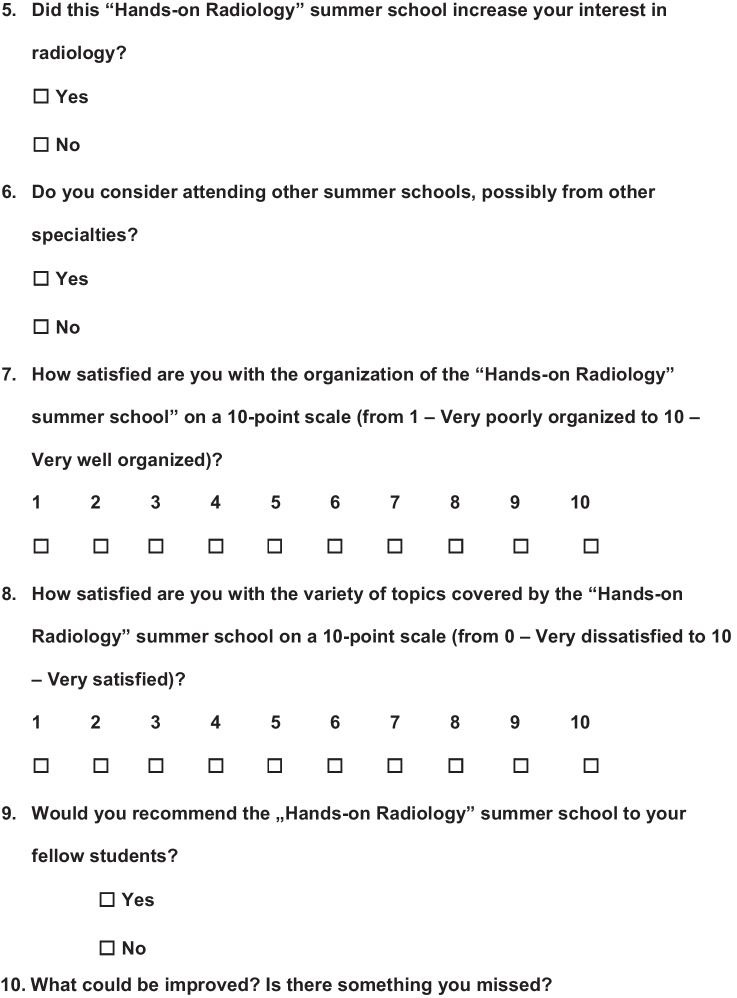

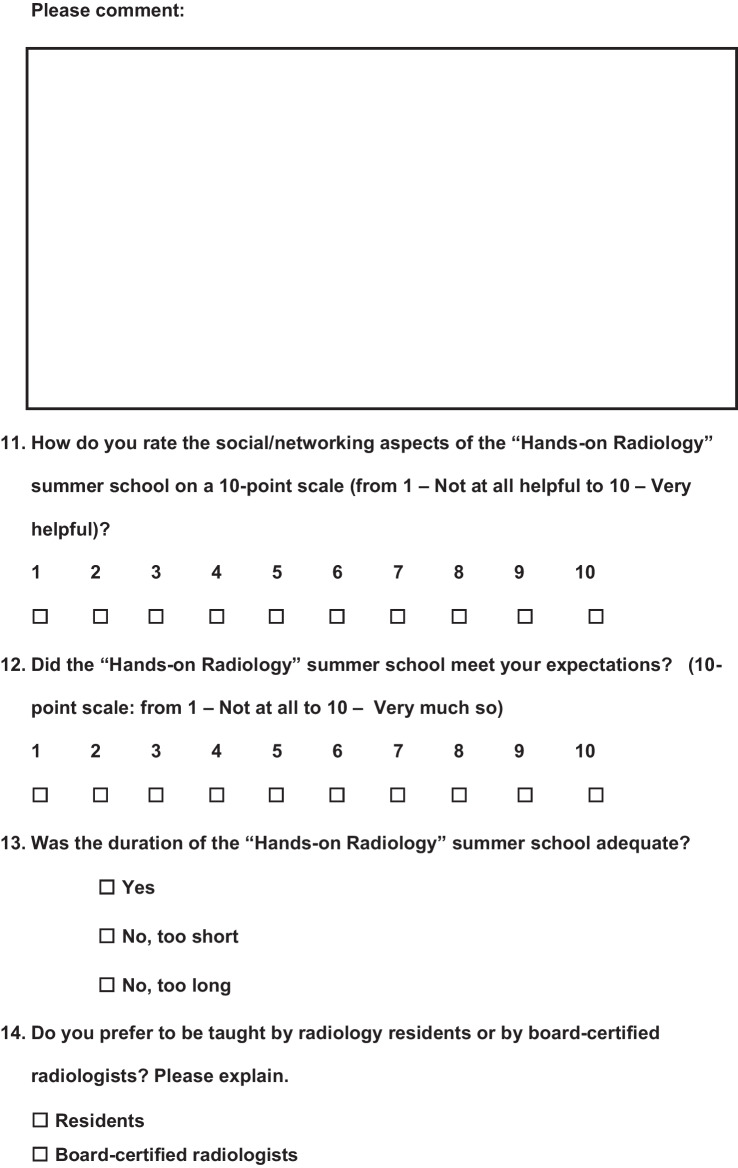

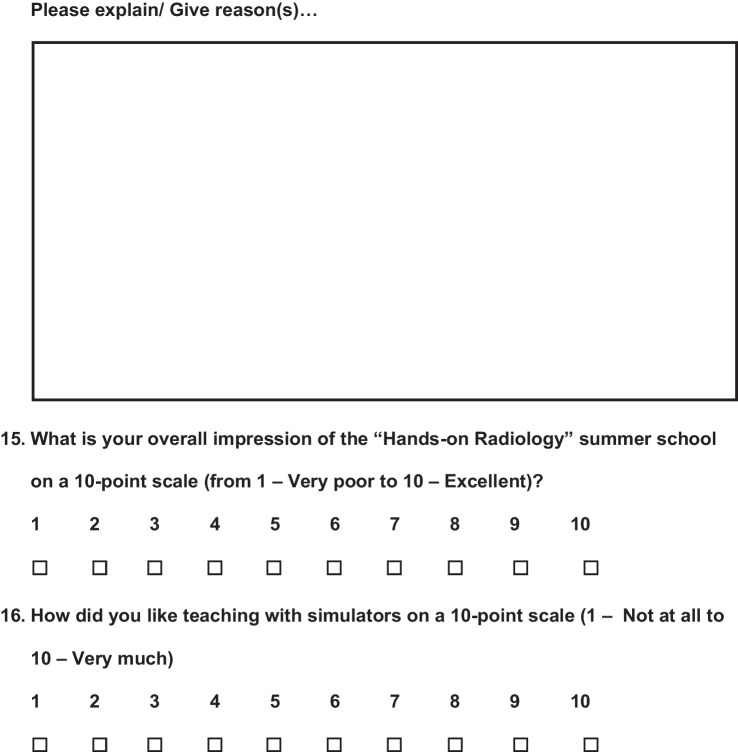
Questionnaire at the end of the Summer School

A voluntary survey was carried out at the beginning of the summer school and at the end. The questionnaire for the first day (day 1) included six questions, of which 2 were multiple choice questions, 3 open comment questions, and 1 10-point scale question (Fig. 1), while the questionnaire at the end (day 3) was longer and consisted of a total of 16 questions including 6 multiple choice questions, 3 open comment questions, and 7 10-point scale questions (Fig. 2). The questionnaires' open comment questions were analyzed separately, and interesting findings are addressed in the Discussion section.

**Fig. 3:**
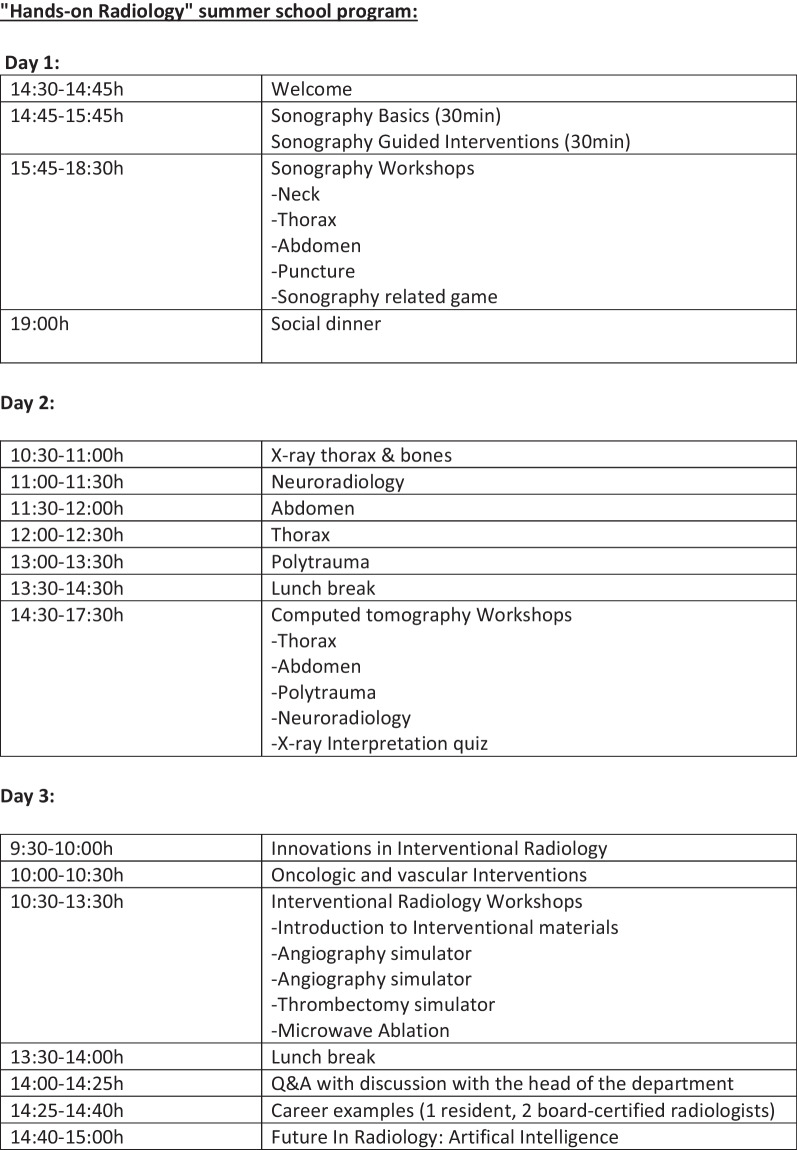
Program

The exclusion criteria for individual questions of the survey were incomplete or inconclusive answers. The summer school program (see Fig. 3) consisted of keynote lectures and workshops in small groups with up to five participants and one tutor. Workshops were based on a small group rotation system to ensure that each group spent the same amount of time at each workshop. The tutors remained at their respective workshop station. The first day started with hands-on ultrasound examinations and US-guided interventions, followed by a social dinner in the evening to promote communication among participating students in a relaxed atmosphere. Students were invited to ask speakers and tutors about their careers and subspecializations and for personal advice regarding radiology in general and career planning. The second day focused on X-ray and computed tomography examinations including intensive small group sessions covering the entire range of diagnostic imaging (e.g., chest/abdominal/trauma and oncological imaging). The third day was dedicated to lectures and workshops on interventional radiology in the morning and career questions and advice in the afternoon. All data were analyzed using Microsoft Excel (Version 16.59).

## Results

### General organization

Overall, 100% (30/30) of the students completed both questionnaires (first and last day questionnaire). The number of different universities of applicants and participants is shown in Table 1.

**Table 1:** General characteristics of applicants and participants

	Applicants	Participants
Total	178	30
Male-to-female ratio	76:102	15:15
Universities	32	21
Average semester	8.4	9.6
Standard deviation	2.5	1.8
Median semester	8	9.5
Interquartile range	3	2.5

Students found out about the summer school mostly through their local student association (36.7%), social media (33.3%), and friends (20.0%) (Table 2). Networking aspects were rated as 8.8/10. Further results of the questionnaires completed on day 1 and day 3 are presented in Tables 2 and 3 (comment sections/questions not shown). The average overall rating of the summer school on a 10-point scale was 9.47. The selection of topics was rated as 9.37 and the duration of two and a half days was mostly seen as sufficient (73.3% (22/30)), while the remaining 26.7% considered the summer school too short. The general organization of the event was assigned a 9.30 rating on a 10-point scale.

**Table 2:** Results of the Questionnaire Day 1

Answer	Yes	Rather yes	Maybe	Rather no	No
*1. Do you consider specializing in radiology?*					
Absolute	15	4	8	3	0
Percentage	50.00%	13.30%	26.70%	10.00%	0.00%

Answer, 10-point scale:	1	2	3	4	5	6	7	8	9	10
*2. How do you rate your level of knowledge in radiology compared to your fellow students on a 10-point scale? (from 1—Very low to 10—Very high)*										
Absolute	0	1	1	2	0	9	11	4	2	0
Percentage	0.00%	3.30%	3.30%	6.70%	0.00%	30.00%	36.70%	13.30%	6.70%	0.00%
Average	6.47									
Standard deviation	1.54									
Median	7									
Interquartile range	1									

Answer	Online	Onsite
*3. What do you prefer: online or onsite teaching?*		
Absolute	2	27
Percentage	6.90%	93.10%

Answer	Student association	Social media	YR homepage/newsletter	Friends	Other
*4. How did you get to know about this summer school?*					
Absolute	11	10	1	6	2
Percentage	36.70%	33.30%	3.30%	20.00%	6.70%

**Table 3:** Results of the Questionnaire Day 3

Answer	More interested	Less interested	No change
*1. Has your attitude towards specializing in radiology changed?*			
Absolute	23	1	6
Percentage	76.70%	3.30%	20.00%

Answer	Yes	Rather yes	Maybe	Rather no	No
*2. Do you now consider specializing in radiology?*					
Absolute	16	6	5	3	0
Percentage	53.30%	20.00%	16.70%	10.00%	0.00%

Answer, 10-point scale	1	2	3	4	5	6	7	8	9	10
*3. How do you rate your level of knowledge in radiology compared to your fellow students on a 10-point scale? (from 1—Very low to 10—Very high)*										
Absolute	0	1	0	1	0	3	6	13	5	1
Percentage	0.00%	3.30%	0.00%	3.30%	0.00%	10.00%	20.00%	43.30%	16.70%	3.30%
Average	7.5									
Standard deviation	1.54									
Median	8									
Interquartile range	1									

Answer	Online	Onsite
*4. What do you prefer: online or onsite teaching?*		
Absolute	1	29
Percentage	3.30%	96.70%

Answer	Yes	No
*5. Did this “Hands-on Radiology" summer school increase your interest in radiology?*		
Absolute	29	1
Percentage	96.70%	3.30%

Answer	Yes	No
*6. Do you consider attending other summer schools, possibly from other specialties?*		
Absolute	29	1
Percentage	96.70%	3.30%

Answer, 10-point scale	1	2	3	4	5	6	7	8	9	10
*7. How satisfied are you with the organization of the “Hands-on Radiology" summer school on a 10-point scale (from 1—Very poorly organized to 10—Very well organized)?*										
Absolute	0	0	0	0	0	0	2	5	5	18
Percentage	0.00%	0.00%	0.00%	0.00%	0.00%	0.00%	6.70%	16.70%	16.70%	60.00%
Average	9.3									
Standard deviation	0.97									
Median	10									
Interquartile range	1									

Answer, 10-point scale	1	2	3	4	5	6	7	8	9	10
*8. How satisfied are you with the variety of topics covered by the "Hands-on Radiology" summer school on a 10-point scale (from 0—Very dissatisfied to 10—Very satisfied)?*										
Absolute	0	0	0	0	0	0	0	4	11	15
Percentage	0.00%	0.00%	0.00%	0.00%	0.00%	0.00%	0.00%	13.30%	36.70%	50.00%
Average	9.37									
Standard deviation	0.71									
Median	9.5									
Interquartile range	1									

Answer	Yes	No
*9. Would you recommend the „Hands-on Radiology" summer school to your fellow students?*		
Absolute	30	0
Percentage	100%	0.00%

Answer, 10-point scale	1	2	3	4	5	6	7	8	9	10
*10. How do you rate the social/networking aspects of the “Hands-on Radiology" summer school on a 10-point scale (from 1—Not at all helpful to 10—Very helpful)?*										
Absolute	0	0	0	0	1	1	3	6	6	13
Percentage	0.00%	0.00%	0.00%	0.00%	3.30%	3.30%	10.00%	20.00%	20.00%	43.30%
Average	8.8									
Standard deviation	1.35									
Median	9									
Interquartile range	2									

Answer	Yes	No, too short	No, too long
*11. Was the duration of the “Hands-on Radiology" summer school adequate?*			
Absolute	22	8	0
Percentage	73.30%	26.70%	0.00%

Answer	Residents	Board-certified radiologists
*12. Do you prefer to be taught by radiology residents or by board-certified radiologists? Please explain*		
Absolute	18	11
Percentage	62.10%	37.90%

Answer, 10-point scale	1	2	3	4	5	6	7	8	9	10
*13. What is your overall impression of the “Hands-on Radiology" summer school on a 10-point scale (from 1—Very poor to 10—Excellent)?*										
Absolute	0	0	0	0	0	0	1	0	13	16
Percentage	0.00%	0.00%	0.00%	0.00%	0.00%	0.00%	3.30%	0.00%	43.30%	53.30%
Average	9.47									
Standard deviation	0.67									
Median	10									
Interquartile range	1									

Answer, 10-point scale	1	2	3	4	5	6	7	8	9	10
*14. How did you like teaching with simulators on a 10-point scale (1—Not at all to 10—Very much)*										
Absolute	0	0	0	0	0	0	1	5	7	17
Percentage	0.00%	0.00%	0.00%	0.00%	0.00%	0.00%	3.30%	16.70%	23.30%	56.70%
Average	9.33									
Standard deviation	0.87									
Median	10									
Interquartile range	1									

### Radiological knowledge and interest

Participants were asked to rate their level of knowledge in radiology compared to their fellow students on a 10-point scale. The median level was 6.47 on day 1 (IQR, 6–7) versus 7.50 on day 3 (IQR, 7–8), corresponding to a median increase of 1.03. Nearly all participants (96.7%, *n* = 29/30) stated that the summer school increased their interest in radiology. On day 3, a high number of participants (76.7%, *n* = 23/30) stated that they were more inclined to specialize in radiology, while only 3.3% (*n* = 1/30) were less inclined, and 20.0% (*n* = 6/30) said that their attitude did not show a relevant change. All participants (100%) would recommend the “Hands-on Radiology” summer school to their fellow students. Practicing with simulators received a high median score of 9.33 on a 10-point scale. In the questionnaire of day 3, 96.7% of participants (*n* = 29/30) said that they prefer onsite teaching. Interestingly, students favored hands-on teaching and lectures by residents rather than board-certified radiologists (62.1 vs. 37.9%).

## Discussion

The main results of our survey among thirty participants of the “Hands-on Radiology” summer school can be summarized as follows: (i) visibility of radiology as a specialization was enhanced as the three-day program increased both interest in and knowledge of radiology and participants’ motivation to consider specialization in radiology, (ii) networking is a major factor during such courses among students, and (iii) students favored onsite teaching, teaching by residents, and connecting with other students and tutors. In addition, the format may be suitable to be implemented on an international level.

Our finding that nearly all participants (96.7%) stated that the summer school increased their interest in radiology is consistent with publications on summer schools for students and young residents from other specialties, which also found an increased interest in their specialty among participants after completion of the program. For example, a study of the orthopedics summer school in Germany found that all participants later worked in orthopedics [6]. In this context, it is worth noting that five of the authors of the present study also attended one of the previous “Hands-on Radiology” summer schools.

Additionally, the students in our survey appreciate being able to practice with interventional radiology simulators, as they provide excellent feedback. A similar result was also reported for a gynecological summer school [5]. Interestingly, students see advantages in being taught by young residents as they seem to be more approachable. Nevertheless, attitudes expressed in comments are more mixed and students see advantages and disadvantages for both residents and board-certified radiologists as teachers. A few participants commented that the preference for certain teachers highly depends on the person’s motivation for teaching and not his or her professional position (resident vs. board-certified radiologist). There are several publications regarding the improvement in radiology teaching; for example, Chew et al. investigated whether more hours of radiology teaching would lead to medical students choosing radiology to become radiologists. They found no correlation with the quantity of radiology teaching, suggesting that quality and “hands-on” experience may be stronger motivators [12, 13]. Interestingly, regarding a gain in knowledge, there were comments from students that they overestimated their knowledge initially when comparing themselves to participants from other universities. A few students also commented that they realized that their knowledge in radiology was lower in direct comparison to participants from other universities. This aspect may be mitigated by a more prominent role of radiology within the national curriculum, which has just been set up in Germany [14].

Web-based teaching and teaching models like flipped classroom became more important in both curricular and extracurricular education during the pandemic over the last years. Nevertheless, nearly all participants preferred onsite teaching (96.7%) to online formats, which is in line with several surveys conducted during the pandemic, showing that students state online learning is not comparable to in-person teaching [15]. In the comment section of our survey, many students said that they are more focused on onsite teaching and that they feel they can better ask questions in person than online.

Our survey has some limitations. Only students from Germany and neighboring countries participated, and their medical education may differ from that of other countries. As we could not accept more than 30 participants to ensure hands-on workshops in small groups, our results and the suggestions made by this selected set of participants may not be representative for all medical students. There is probably a selection bias for motivated students who were chosen to participate because of their motivational letters. Additionally, as the students still needed to pay for traveling and accommodation, we may have excluded less well of students who did not apply for financial reasons. This bias needs to be overcome in future studies. The orthopedics summer school already mentioned above could be a shining example where traveling and accommodation costs were reimbursed [6]. In summary, our “Hands-on Radiology” summer school was a real success on a national level. In addition to participating students’ enthusiasm for the subject of radiology, early professional networking among participants is also a great advantage of this event. However, this potential, whether professional or scientific, can be enhanced further by organizing such events on an international level.

## Conclusion

In conclusion, our survey of the 2022 “Hands-on Radiology” summer school shows that this form of onsite teaching remains a useful tool not only to improve radiology knowledge but also to strengthen interest in radiology among undergraduate medical students.

## Data Availability

The individual questionnaires are not shared to keep every participant anonymous; the compiled data can be found in the tables attached to the paper.
